# Strategies for carbohydrate model building, refinement and validation

**DOI:** 10.1107/S2059798316016910

**Published:** 2017-02-01

**Authors:** Jon Agirre

**Affiliations:** a York Structural Biology Laboratory, Department of Chemistry, University of York, York YO10 5DD, England

**Keywords:** carbohydrates, glycosylation, conformation, restraints, validation

## Abstract

Sugars are the most stereochemically intricate family of biomolecules and present substantial challenges to anyone trying to understand their nomenclature, reactions or branched structures. Current crystallographic programs provide an abstraction layer allowing inexpert structural biologists to build complete protein or nucleic acid model components automatically either from scratch or with little manual intervention. This is, however, still not generally true for sugars. The need for carbohydrate-specific building and validation tools has been highlighted a number of times in the past, concomitantly with the introduction of a new generation of experimental methods that have been ramping up the production of protein–sugar complexes and glycoproteins for the past decade. While some incipient advances have been made to address these demands, correctly modelling and refining carbohydrates remains a challenge. This article will address many of the typical difficulties that a structural biologist may face when dealing with carbohydrates, with an emphasis on problem solving in the resolution range where X-ray crystallography and cryo-electron microscopy are expected to overlap in the next decade.

## Abstract 2

This article addresses many of the typical difficulties that a structural biologist may face when dealing with carbohydrates, with an emphasis on problem solving in the resolution range where X-ray crystallography and cryo-electron microscopy are expected to overlap in the next decade.

## 1. Cinderella’s coach may not be ready yet   

**Figure 1:**
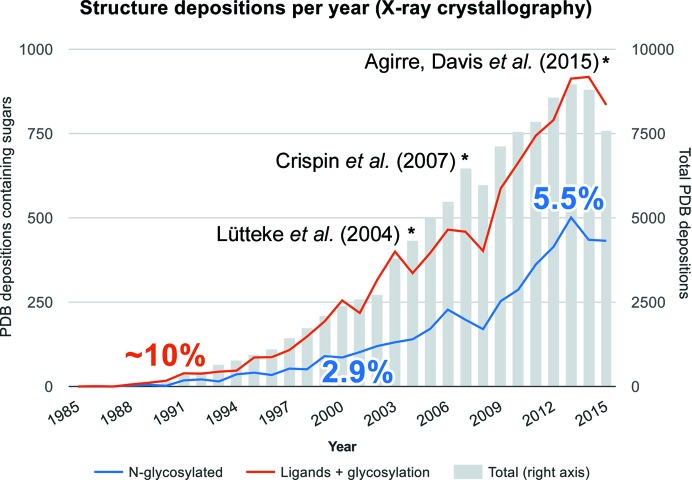
Deposition rate of glycoproteins and protein–sugar complexes. This graph was produced using the publicly available search functions provided by the RCSB PDB (Bernstein *et al.*, 1977 ▸), restricting each query to crystallographic structures. Structures containing saccharides were selected by the different ‘saccharide’ chem_comp codes (represented by a red line on the graph), and structures containing N-­glycosylation were selected by the ASN_ND2_-NAG_C1_ LINK record (represented by a blue line); the latter figures do not reflect the total number, as at least 16 structures were found to have incorrect ASN_OD1_-NAG_C1_ LINK records. The total numbers of PDB structures per year (grey bars) have been plotted on a 1/10 scale (right axis) to make the 10% proportion stand out.

The author does not intend to rewrite fairytale canon, but to bridge the 15-year gap between the biotechnological breakthroughs highlighted in the now classic *Science* editorial (Hurtley *et al.*, 2001 ▸) that the title of this section alludes to and the current state of the art in structural glycobiology. For the past 35 years and apparently conforming to some kind of law, carbohydrate-containing structure depositions, signified by a red line in Fig. 1 ▸, have steadily matched 10% of the annual total. However, the balance within this seemingly fixed percentage has strikingly changed in the past decade: glycosylation, which groups a number of post-translational and co-translational covalent modifications of proteins with sugars, has become increasingly frequent. N-glycosylation alone (blue line in Fig. 1 ▸), the most frequently reported type, has increased from 2.9% in 2000 to 5.5% of the total in 2013. While ligand carbohydrates continue to be the focus of many biotechnological and biomedical studies, it would seem that the contribution of glycosylation to eukaryotic protein folding, stability and function is progressively taking the spotlight. This is already having implications: while the number of ligand sugars per structure will usually be within one to a couple of dozen at most, heavily glycosylated structures are becoming more frequent and can contain over 100 monosaccharides each (see, for example, Agirre *et al.*, 2016 ▸; Gudmundsson *et al.*, 2016 ▸; Stewart-Jones *et al.*, 2016 ▸), increasing the number of deposited monosaccharide models per year. Cryo-electron microscopy (cryo-EM), a structural technique that does not depend on the ordered packing of particles into crystals, is not vulnerable to the deleterious effects that external glycans may have (Pallesen *et al.*, 2016 ▸), and thus is expected to contribute strongly to this trend in forthcoming years.

Regrettably, the purely methodological side of structural glycobiology has not kept up with the experimental advances: more, but not better, carbohydrate structures are being deposited. Indeed, a number of concerns have been raised with respect to the validity or meaningfulness of the sugars in the PDB. The work of Lütteke *et al.* (2004 ▸) was the first to highlight that as many as 30% of the deposited entries contained nomenclature errors ranging from residue-naming problems to linkage specifications (for example, incorrect distances, chemically impossible valences or the wrong choice of leaving groups). A few years later, Crispin *et al.* (2007 ▸) raised their voice to require that structural glycobiologists honour the prior knowledge of the sequence and structure of N-linked glycans when modelling carbohydrate structures at low resolution. This correspondence was met with a letter from the PDB in which they acknowledged the issue and highlighted the availability of tools such as *PDB-CARE* (Lütteke & von der Lieth, 2004 ▸) for nomenclature validation. More recently, another study reported a worrying situation affecting ring conformation (Agirre, Davies *et al.*, 2015 ▸), using N-glycan-forming d-pyranosides as an example, although the results clearly extend not only to all pyranose sugars but to every ligand with a saturated six-membered ring. In general, the software tools that deal admirably with proteins (Murshudov *et al.*, 2011 ▸; Adams *et al.*, 2011 ▸; Emsley *et al.*, 2010 ▸; Blanc *et al.*, 2004 ▸; Brünger *et al.*, 1998 ▸), and nucleic acids to some extent, appear to have problems handling ligands at lower resolution (Reynolds, 2014 ▸; Liebeschuetz *et al.*, 2012 ▸; Perola & Charifson, 2004 ▸), with thousands of structures showing angle and torsional strains that cannot be supported unequivocally by the experimental data. Pyranosides, and indeed all saturated cyclic compounds, might not necessarily show such strains, but can be spuriously locked into secondary energy minima (typically boat conformations) that may only show transannular strain (Agirre, Davies *et al.*, 2015 ▸) after rounds of model refinement against low-resolution data. While these conformations do appear in nature, for most sugars they arise from a conformational transition that requires a high energy barrier to be overcome. Traversing such a barrier almost exclusively requires enzyme-assisted catalytic events (Davies *et al.*, 2012 ▸), thus making any sugar model in a high-energy conformation a chemical statement in itself. As reported by Agirre, Davies *et al.* (2015 ▸), the occurrence of high-energy conformations in N-glycan-forming d-pyranosides solved at high resolution is correlated with errors introduced during model building and refinement. At low resolution (worse than 1.5 Å), these are augmented by the challenges inherent to interpreting broad and poorly structured electron density, either visually (an incorrect choice is made by the crystallographer) or computationally (the refinement software chooses one of many, equally wrong, minima owing to a deficit in restraints). New carbohydrate-specific methods are essential to address these problems and the structural biology community needs to be persuaded to embrace this good practice, otherwise these problems will propagate to a new level with the rise of cryo-EM, which is now consistently delivering structures in precisely the limited resolution range where most conformational anomalies occur.

## 2. Many possibilities, different probabilities   

**Figure 2:**
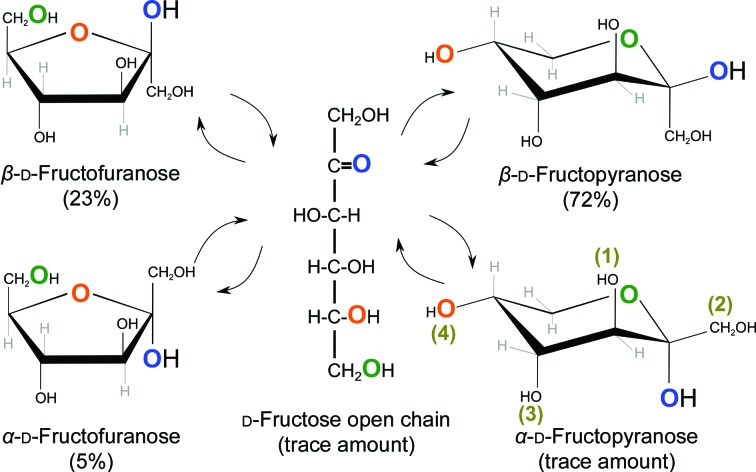
Interconversions between open-chain and cyclic forms of d-fructose. A furanose ring (on the left) is formed after the 5-hydroxyl (O atom in orange) performs a nucleophilic attack on the ketone (carbonyl containing the O atom in blue). This results in two anomeric configurations (α or β, resulting from the blue O atom lying on the lower or upper side of the ring. respectively), as the ketone C atom is *sp* ^2^-hybridized and thus planar, and the attack can be performed from either side of the plane. The same holds true for pyranose-ring formation, except that now it is the 6-hydroxyl (O atom in green) which attacks the ketone. A similar mechanism occurs in aldoses (*e.g.* d-glucose or d-galactose), where the 4- and 5-hydroxyls attack the aldehyde group in position 1 to form furanose and pyranose rings, respectively. Numbers in gold denote all of the potential positions that substituents can adopt in a pyranose ring (1, up and axial; 2, up and equatorial; 3, down and axial; 4, down and equatorial).

Sugars come in many stereochemistries, configurations, forms and conformations (for a concise introduction, see Bertozzi & Rabuka, 2009 ▸). In an enzyme-free reaction (usually catalysed by a dilute base or acid), they may interconvert from an open-chain form to a furanose cyclic form (five-membered saturated ring) or a pyranose cyclic form (six-membered saturated ring). These transitions depend on the stability of each form, and all forms can co-exist in solution, although conversion from the cyclic form to the open chain requires a free hemiacetal (if the sugar is an aldose) or a hemiketal (if the sugar is a ketose) group, *i.e.* that the sugar is not linked to another through C1 (C2 if the sugar is a ketose). Stereochemistry defines the sugar, and particular attention must be paid to two key conventions: absolute configuration and anomeric configuration. The absolute configuration of a monosaccharide, identified by a small capital d or l, is denoted by the configuration of the stereocentre furthest away from the anomeric C atom (usually referred to as the configurational atom; see Fig. 2 ▸, substituent in magenta colour; in the open-chain form right indicates *dextro* and left indicates *laevo*; in the cyclic structures up indicates *dextro* and down indicates *laevo*). With every cyclization, a choice of anomeric configuration is made based on the stereochemical relationship of the resulting hydroxyl group with respect to the anomeric reference atom, which will be the configurational atom except in some special cases (*e.g.* sialic acids), where multiple configurational prefixes are indicated. These configurations, termed anomers, are denoted as α (different stereochemistry at both stereocentres) or β (the same stereochemistry), typically involving comparison of the position (up, down) of the C1 hydroxyl group (C2 in ketoses) with that of the C atom linked to C5 (C6 in ketoses) for the most common monosaccharides. The interconversion between two anomeric forms is called mutarotation and is illustrated in Fig. 2 ▸, which has been annotated with the proportions determined experimentally for d-fructose (a ketose) by Flood *et al.* (1996 ▸). These proportions can help us to understand how stable each form is. The different anomeric configurations affect this stability, as the torsional strain around the link from the anomeric centre to the adjacent C atom will differ. In order to minimize such strain, the conformation of the substituents when viewed across such a link should be staggered (*i.e.* the substituents of one C atom are interleaved with those of the other C atom) rather than eclipsed, which would lead to van der Waals (vdW) repulsion. As mutarotation requires the sugar to pass through the open-chain form, only those monosaccharides that are either free or at the reducing end (see below) of a polysaccharide will be able to interconvert between anomeric forms.

**Figure 3:**
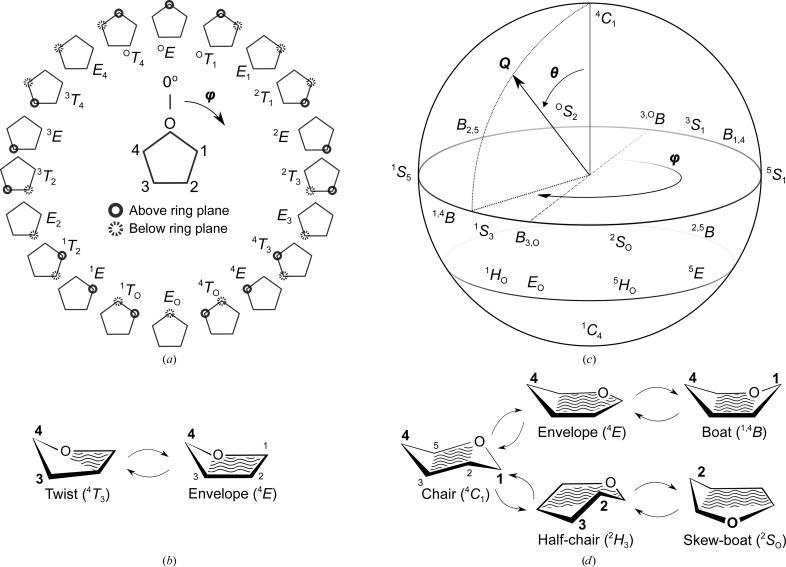
Conformational interconversions. According to IUPAC carbohydrate nomenclature (McNaught, 1997 ▸), the different conformations are identified by an italic capital letter, chair (*C*), envelope (*E*), boat (*B*), skew-boat (*S*), half-chair (*H*) and twist (*T*), with the atoms on the upper or lower side of the main ring plane in superscript and subscript lettering, respectively. Wavy lines identify those atoms that are roughly coplanar (*i.e.* forming the main plane) in that particular conformation. Here, the different conformations are drawn as a function of the Cremer–Pople puckering parameters (Cremer & Pople, 1975 ▸). (*a*, *b*) Pseudo-rotational itinerary for furanoses and possible conformations. Furanoses are able to adopt twist and envelope conformations, with a very small energy barrier separating them. O atoms, which are assumed to be located at the top vertex in the pentagons, have been omitted from this diagram for reasons of clarity. In addition, the diagram does not show the total puckering amplitude (*Q*). (*c*, *d*) Cremer–Pople sphere describing the conformational itineraries for pyranoses and possible conformations. In order to convert the chair conformation of a pyranose ring to a boat conformation, both of which typically sit at energy minima, with the chair being the more favourable, the ring must pass through envelope or half-chair conformations which, having eclipsed substituents and considerable angle strain, require a considerable energetic investment. In context, these energy barriers are usually proportional to the cost of breaking three or four hydrogen bonds in peptides (Sheu *et al.*, 2003 ▸; Davies *et al.*, 2012 ▸).

Cyclic monosaccharides, like all other saturated rings, can be found in a number of conformations with different free energies. Furanose rings can adopt twist (*e.g.* ^O^ *T*_4_, which denotes a twist with the endocyclic O atom positioned on the upper side of the ring and the fourth carbon on the lower side) and envelope conformations (*e.g.* ^4^ *E*; see Fig. 3 ▸), with very little difference in terms of free energy between them (around 1 kcal mol^−1^ based on the results obtained for cyclopentane; Lightner & Gurst, 2000 ▸); although there is very low angle strain, ring puckering helps to relieve some of the more critical torsional strain produced by clashes between substituents. In contrast, pyranose rings do have clear conformational preferences owing to the possibility of reaching the optimal 60° angle between exocyclic atoms, thus relieving much of the potential torsional strain. Pyranose rings can adopt chair (two possible chairs, ^4^ *C*_1_ and ^1^ *C*_4_; refer to the legend of Fig. 3 ▸ for an introduction to the IUPAC conformational nomenclature), half-chair (*e.g.* ^2^ *H*_3_ in Fig. 3 ▸), envelope (*e.g.* ^4^ *E* in Fig. 3 ▸), boat (*e.g.* ^1,4^ *B*, with both C atoms 1 and 4 positioned on the upper side of the ring) and skew-boat conformations (*e.g.* ^2^ *S*_O_ in Fig. 3 ▸), and their interconversion requires an itinerary such as that pictured in Fig. 3 ▸. A chair is always preferred by saturated rings, as it allows the aforementioned optimal 60° angle between substituents. Because the cyclization of d-sugars and l-sugars require specular movements, d-sugars adopt a ^4^ *C*_1_ conformation, whereas l-sugars often find their energy minimum in a ^1^ *C*_4_ conformation, although exceptions do occur whenever the configurational atom is not the last ring C atom. The energy barriers separating each conformation are high, and hence conformational transitions typically require the presence of a catalyst, usually a carbo­hydrate-active enzyme. These enzymes, which have been categorized in the Carbo­hydrate-Active enZYmes database, or CAZy (Lombard *et al.*, 2014 ▸), often distort sugar substrates to achieve optimal orbital overlap in order to perform reactions such as hydrolysis (breakage of the polysaccharide chain), glycoside transfer from an activated nucleotide-sugar (glycosyl donor) or isomerization (*e.g.* the transformation of d-glucose into d-fructose; one stereocentre fewer owing to the formation of an achiral keto group) and epimerization (configurational change at just one stereocentre, *e.g.* transformation of d-glucose into d-mannose).

The required energies and conformational itineraries for enzyme/sugar reactions can be successfully analysed with a hybrid QM/MM metadynamics approach (Laio & Parrinello, 2002 ▸). Its successes in studying glycosidases have been reviewed by Davies *et al.* (2012 ▸), and the field is now also making significant progress towards understanding glycosyltransferases (Ardèvol *et al.*, 2016 ▸). These studies explore the conformational landscape of monosaccharides in terms of the Cremer–Pople puckering coordinates (Cremer & Pople, 1975 ▸), as depicted in Fig. 3 ▸. Two angles, φ and θ (just φ for furanoses) describe which atoms deviate from the mean ring plane, and a puckering amplitude (*Q*) describes how much these atoms move away from this mean plane. In addition, a histogram of values for these puckering coordinates can be derived from metadynamics studies (Iglesias-Fernández *et al.*, 2015 ▸; Ardèvol *et al.*, 2010 ▸; Biarnés *et al.*, 2007 ▸) and has been implemented as a conformational validation criterion in the process of model building with the *Privateer* software (Agirre, Iglesias-Fernández *et al.*, 2015 ▸) which, along with other information, produces the Cremer–Pople parameters and IUPAC conformation designators for most types of sugar in the PDB Chemical Component Dictionary (PDB_CCD_).

**Figure 4:**
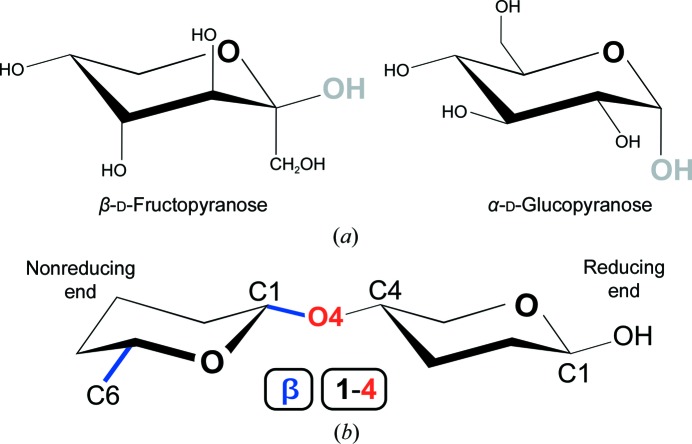
Making linkages. (*a*) Leaving groups. Leaving groups which abandon the reducing sugar during the linkage reaction are depicted in grey. H atoms have been omitted for reasons of clarity. (*b*) Linkage nomenclature. A schematic representation of a glycosidic linkage [the simplified monosaccharides are unrelated to those in (*a*)] is shown. Atoms are referred to by their PDB_CCD_ nomenclature, and those groups responsible for linkage nomenclature have been colour-coded: blue, the configuration of the newly linked O4 (which substitutes O1 from the leaving group) with respect to the absolute stereochemistry as determined by C6 marks the linkage stereochemistry (β); red, the order of the bond (1–4) indicates that the linkage is a glycosidic bond between C1 from the sugar on the left and O4 from the sugar on the right. If the sugar on the left was a ketose, for example d-fructose, the linkage would be signified as β2–4, as the anomeric C atom would be C2 (see Fig. 2 ▸).

Linkages, henceforth referred to as glycosidic bonds, can be produced enzymatically by glycosyltransferases with either inversion or retention of the anomeric configuration. In these reactions, an acetal bond is formed after a nucleophilic displacement of the leaving group at the reducing end of a monosaccharide (grey hydroxyls in Fig. 4 ▸) by a neighbouring alcohol (OH, which will result in ‘O4’ in Fig. 4 ▸, in analogy to crystallographic modelling). This concept is of great importance for the correct generation and recognition of bonds in crystallographic software, as the atom that has to be removed must always be the leaving group. In the example shown in Fig. 4 ▸, a new link description (see below) has to be generated between O4 (which substitutes the leaving group) and C1 (the anomeric C atom from the sugar on the left). In case of uncertainty, it is always worth checking the chemical details of a sugar in the PDB_CCD_, where there is an entry identifying the leaving atom. When glycosidic bonds are established in this way, the resulting polysaccharide will have a reducing end (free hemiacetal or hemiketal; on the right in Fig. 4 ▸) and a nonreducing end (left of Fig. 4 ▸). However, if a glycosidic bond is established between two anomeric C atoms, for example sucrose (α-d-glucopyranose linked 1–2 to β-d-fructofuranose), the resulting disaccharide will be a nonreducing disaccharide. In contrast to the lability of the hemiacetals (aldoses) and hemiketals (ketoses), the acetal and ketal bonds are very stable and breaking them usually requires either an acid- or enzyme-catalysed reaction. Such enzymes are termed glycoside hydrolases and, like glycosyl transferases, they can operate with either inversion or retention of the anomeric configuration.

**Figure 5:**
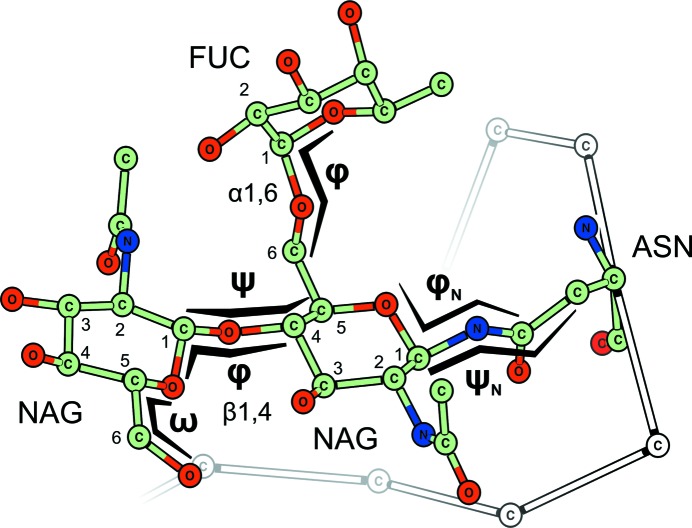
Understanding link torsions. In analogy to how the peptide-bond conformation is evaluated in proteins, glycosidic bonds can also be described in terms of torsions. These have been denoted in lowercase Greek letters in order to avoid confusion with the Cremer–Pople parameters (Cremer & Pople, 1975 ▸), and match the nomenclature as reviewed by Lütteke (2009 ▸) and used by the *CARP* server (Lütteke *et al.*, 2005 ▸, 2006 ▸) as well as *Privateer* (Agirre, Iglesias-Fernández *et al.*, 2015 ▸). Some of these torsion angles are expected to have predictable values as they involve an *sp* ^2^-hybridized C atom, *e.g.* ψ_N_. This figure was generated with *CCP*4*mg* (McNicholas *et al.*, 2011 ▸).

The conformation of the glycosidic bond can be described in terms of torsions, following a convention reviewed by Lütteke (2009 ▸). This convention, depicted in Fig. 5 ▸, may be used to compare torsional data with existing structures using the *CARP* server (Lütteke *et al.*, 2005 ▸), and has been adopted by other programs such as *Privateer* (Agirre, Iglesias-Fernández *et al.*, 2015 ▸). As for ring conformations, excessive torsional strain is not frequent in glycosidic bonds and should be supported by a good fit to the electron density, which is typically assessed by a local correlation metric such as the real-space correlation coefficient (RSCC), which is part of all crystallographic fitting and analysis software packages, as reviewed and expanded by Tickle (2012 ▸).

Interaction of sugars with proteins fall into three general types. In decreasing order of strength, they can firstly be covalently linked to proteins, as in the different forms of glycosylation (see below), secondly they can bind to electronegative atoms in neighbouring residues *via* hydrogen bonds (Fernández-Alonso *et al.*, 2012 ▸), and thirdly they can interact through their apolar face with aromatic residues such as tryptophan (either of the two rings or both), tyrosine and, less frequently, phenylalanine and histidine (Hudson *et al.*, 2015 ▸). While hydrogen bonds have a more active role in structural reinforcement and recognition processes in N-linked and O-linked glycans, aromatic residues are usually involved in the binding of sugars to the active sites of carbohydrate-active enzymes by providing a hydrophobic surface.

### 2.1. In practical terms   

Sugars can interconvert from the open chain to the cyclic form and will often slowly mutarotate as free monosaccharides in solution or at the reducing end of a polysaccharide chain. Most d-sugars will always appear in a ^4^ *C*_1_ conformation and l-sugars in a ^1^ *C*_4_ conformation. Any deviation from this must be supported by the experiment and ideally be reported as a conformational outlier in the crystallographic Table 1 ▸, in the same way that amino-acid main-chain outliers are reported for the protein, as proposed by Ramachandran *et al.* (1963 ▸). Linkages are created by substituting the leaving group of one sugar by the O atom of a neighbouring hydroxyl group (or alternatively an S atom from a thiol group). By convention, this behaviour must be mimicked when linking an atomic model, as programs will not necessarily recognize and assign chirality properly. In analogy to how the peptide-bond conformation is analysed, a convention is required to describe and validate glycosidic linkages in terms of torsions.

## 3. Glycosylation: an underdog goes mainstream   

A number of co-translational and post-translational covalent modifications of protein residues with carbohydrates are categorized according to the glycosylation type. These modifications are not *per se* encoded in genomes, although the modified amino acids may conform to a sequence motif, but instead are fully dependent on the available glycosyltransferases and glycan-processing enzymes (Rini *et al.*, 2009 ▸). Hence, the structural possibilities are limited and usually particular to the expression system used. Based on a genomic analysis, it has been estimated that more than 50% of human proteins are glycosylated (Apweiler *et al.*, 1999 ▸).

The most frequent form is N-glycosylation (N-glycans), which involves the post-translational modification of an asparagine residue (Asn) adhering to the sequence motif Asn-*X*-Ser/Thr (N-glycan recognition site, or sequon) with an *N*-acetyl β-d-glucosamine (GlcNAc) sugar, linked through the N atom of the side chain (ND2 in PDB nomenclature) by a multiprotein complex named oligosaccharyl transferase (OTase). This modification is only possible with the β anomer of GlcNAc. The production of N-glycans begins in the endoplasmic reticulum with the *en bloc* transference by OTase of a common dolichol-linked precursor oligosaccharide to a nascent polypeptide, forming a proto-glycoprotein (Dempski & Imperiali, 2002 ▸), which will benefit from the structural reinforcement that the glycans provide (Helenius & Aebi, 2004 ▸). This oligosaccharide has a defined chemical structure (d-glucose_3_, d-mannose_9_, *N-*acetyl β-d-glucosamine_2_), and is trimmed and modified later as required. Its most common form after the initial trimming is called high-mannose (d-mannose_9_, *N-*acetyl β-d-glucosamine_2_). Some glycans remain in this form, but many undergo further processing in the Golgi (Varki, Esko *et al.*, 2009 ▸).

**Figure 6:**
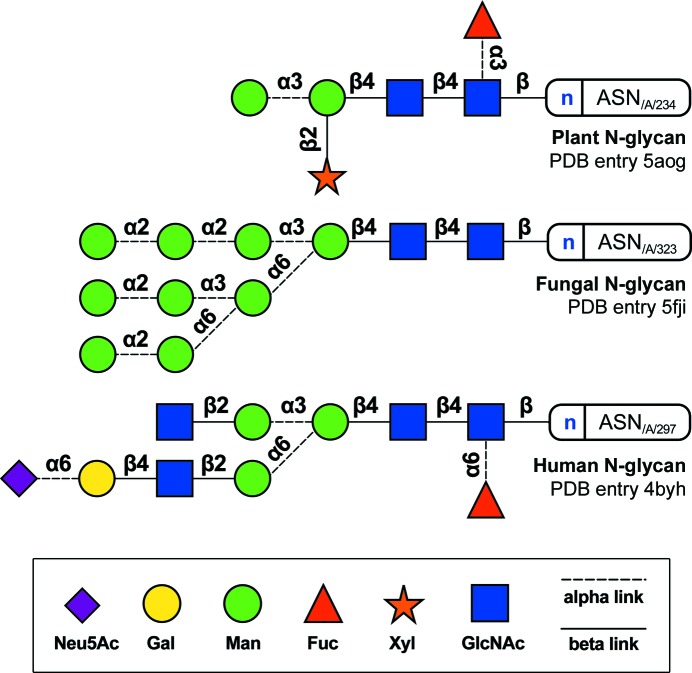
Examples of N-linked glycosylation. Top, plant N-glycans typically show α1–3 core-linked fucose and β1–2 xylose linked to the first mannose sugar. In the figure, a diagram of one of the glycans found in a haem peroxidase from sorghum (PDB entry 5aog; Nnamchi *et al.*, 2016 ▸). Middle, a complete, unprocessed high-mannose N-glycan linked to a glycosyl hydrolase enzyme from the fungus *Aspergillus fumigatus* (PDB entry 5fji; Agirre *et al.*, 2016 ▸). Bottom, a sialylated N-glycan linked to an Fc fragment from a human antibody (PDB entry 4byh; Crispin *et al.* (2013 ▸). Human glycans, and also mammalian glycans in general, may display an α1–6 core-linked fucose. All diagrams and legends were generated with *Privateer* (Agirre, Iglesias-Fernández *et al.*, 2015 ▸). For more examples of glycans, refer to the complete overview of N-glycan structures published by Stanley & Cummings (2009 ▸).

There is a limited range of trimming and transference enzymes, and in addition a limited range of building blocks which they can handle (Rini *et al.*, 2009 ▸). Hence, a reduced set of graphical symbols can be used to represent their stereochemistry, derivatives and anomericity. This was originally proposed by Kornfeld *et al.* (1978 ▸), standardized in *Essentials of Glycobiology* (Varki *et al.*, 1999 ▸), and subsequently improved (Varki, Cummings *et al.*, 2009 ▸; Varki *et al.*, 2015 ▸) while simultaneously incorporating interesting elements from other nomenclature systems such as the Oxford system (Harvey *et al.*, 2009 ▸). The *Essentials of Glycobiology* (hereafter termed ‘Essentials’) nomenclature encodes glucose stereochemistry in blue, galactose in yellow and mannose in green, while signifying unmodified hexoses (six-carbon sugars) by a circle, *N*-acetylhexosamines by a square, hexosamines by a square divided diagonally and acidic sugars by a diamond. For complete correspondence between the Essentials nomenclature and the PDB_CCD_ three-letter codes used by the structural biology community, and a three-dimensional extension to this nomenclature, refer to McNicholas & Agirre (2017 ▸). A number of expression system-dependent N-glycosylation examples can be seen in Fig. 6 ▸, which has been composed using the latest Essentials nomenclature as produced by the CCP4 sugar validation and analysis program *Privateer* (Agirre, Iglesias-Fernández *et al.*, 2015 ▸). Additionally, thanks to the *GlycanBuilder* graphical software (Damerell *et al.*, 2012 ▸, 2015 ▸), it is possible to interrogate mass-spectrometry databases such as UniCarbKB (Campbell *et al.*, 2014 ▸) and more recently glycosciences.de (Loss & Lütteke, 2015 ▸; Lütteke *et al.*, 2006 ▸) using the familiar Essentials nomenclature to browse for experimental evidence of the occurrence of a particular glycan in a particular expression system. N-glycan structures have been reviewed in detail in Stanley & Cummings (2009 ▸).

A second type of covalent modification is O-glycosylation, which most frequently involves a serine or threonine residue being modified with *N*-acetyl α-d-galactosamine (GalNAc). Other modifications include O-linked mannosylation, fucosyl­ation, xylosylation, galactosylation, glucosylation or, notably, intracellularly O-linked *N*-acetyl β-d-glucosamine. O-glycosylation is less frequently modelled than N-glycosylation, and is less well understood. To date, it accounts for less than 1% of the glycosylated structures deposited in the PDB.

Glycosylation has been historically overlooked and largely ignored. However, especially in the last decade, it has become increasingly evident that the interactions provided by covalently linked glycans are not only of structural importance but also of great functional relevance (Sinclair & Elliott, 2005 ▸). N-linked glycans play a major recognition role in antibodies (Varki & Lowe, 2009 ▸), which depend on their three-dimensional conformation and hydrogen-bond interactions for their beneficial therapeutic effects. In addition, O-GalNAc glycans linked to mucins have implications in many signalling and communication processes, including cancer, with a central role in metastasis formation (Pinho & Reis, 2015 ▸).

As Fig. 1 ▸ reveals, the structural biology of glycosylation is finally taking off.

### 3.1. In practical terms   

Since glycans are linked to protein *via* the anomeric C atom, they are inherently nonreducing and once they have formed mutarotation is absolutely out of the question. Glycans that are exposed to the solvent can be expected to interfere with crystallization by hindering the formation of crystal contacts owing to their mobility [see Wyss *et al.* (1995 ▸) for an NMR ensemble of a glycoprotein, also represented in this issue (McNicholas & Agirre, 2017 ▸)], and it may be viable to remove them without loss of activity should the first crystallization trials fail, provided that the protein is still able to fold. This can be performed enzymatically, for example with endoglycosidase H. This should not be a problem in single-particle cryo-EM, as any flexible external glycans will simply be averaged out during the image-reconstruction process.

Glycans should be modelled based on prior knowledge of their structure. This can be checked by accessing mass-spectrometry databases, but also by looking at PDB structures, provided that they have a good fit to the experimental data. It is possible to use the Glycoblocks representation (McNicholas & Agirre, 2017 ▸) within *CCP*4*mg* (McNicholas *et al.*, 2011 ▸) to visually analyse N- and O-glycan structures in two dimensions and three dimensions using the Essentials nomenclature, which in addition will identify any potential errors, as the two-dimensional diagrams embed the validation information computed by *Privateer* (Agirre, Iglesias-Fernández *et al.*, 2015 ▸).

## 4. Dictionaries for sugars   

**Figure 7:**
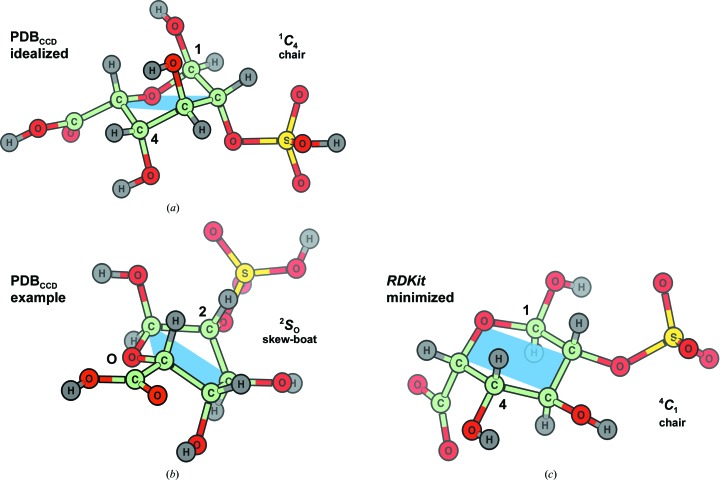
Idealized and example coordinates for the PDB_CCD_ entry IDS (2-*O*-sulfo α-l-iduronic acid) and their comparison with a minimal energy conformer calculated by torsional exploration and minimization with *RDKit*. The blue area denotes those atoms which lie roughly in a plane, making it easier to identify the ring conformation. Top, the biologically relevant ^1^ *C*_4_ conformer, as stored in the PDB_CCD_ idealized coordinates. Despite showing repulsion between axial substituents, this chair conformation is the only feasible conformation, as converting it into the slightly more favourable ^4^ *C*_1_ chair would require a considerable energetic investment. Middle, example coordinates as determined by NMR (Mulloy *et al.*, 1993 ▸). This conformer is in a high-energy conformation and does not match any of the available high-resolution crystallographic structures. Bottom, a ^4^ *C*_1_ chair conformer obtained by torsional exploration with *RDKit* (Landrum, 2016 ▸). The aforementioned energy barrier is artificially circumvented by exploring different combinations of torsions. This is the absolute minimal energy conformation, but one that is not attainable without external intervention. This figure was generated with *CCP*4*mg* (McNicholas *et al.*, 2011 ▸).

All major macromolecular crystallographic refinement packages use a monomer dictionary library to organize prior chemical knowledge in the form of geometric restraints. Many of them have extended or have used at some point, with manual curation, the initial CCP4 monomer library of Vagin *et al.* (2004 ▸). This initiative produced, using *LIBCHECK* (Vagin *et al.*, 2004 ▸) with irregular results (see below), geometric targets consistent with Engh & Huber (1991 ▸) from all of the entities (henceforth monomers) in the PDB Chemical Component Dictionary (PDB_CCD_) at that point in time. The PDB_CCD_ is the place of reference for obtaining codes, names and chemical descriptions of the very building blocks that structural biology relies upon: monomers. These are stored in files containing a topological description of the monomer along with example Cartesian coordinates, extracted from a deposited experimental structure, and/or computationally idealized coordinates (Westbrook *et al.*, 2015 ▸). Both sets are available from the PDB_CCD_ in SDF format (Molecular Design Ltd,), and can be inspected with either *PyMOL* (v,8; Schrödinger) or *UCSF Chimera* (Pettersen *et al.*, 2004 ▸). While the two sets of coordinates should always be representative and almost identical for simple monomers, discrepancies do occur. Calculating the minimal energy conformation for larger structures, for example polysaccharides, with many degrees of freedom can be very expensive in computational terms, and can fail to replicate what is found in nature. Monosaccharides, like other saturated rings, pose particular problems for minimization, thus the results need experimental validation, ideally with a high-resolution small-molecule structure. One such example is the PDB_CCD_ entry IDS (2-*O*-sulfo-α-l-iduronic acid), an l-sugar, which includes a ^1^ *C*_4_ chair conformer in the idealized coordinates (Fig. 7 ▸, top panel) and a high-energy ^2^ *S*_O_ skew-boat conformer that was determined by solution NMR (Mulloy *et al.*, 1993 ▸) in the example coordinates (Fig. 7 ▸, middle panel). Furthermore, a different answer, a lowest-energy ^4^ *C*_1_ chair, is obtained when generating a conformer from its SMILES string by sampling the torsional space of the monomer randomly with *RDKit* (Landrum, 2016 ▸) followed by energy minimization (Fig. 7 ▸, bottom panel). So the question for the user is ‘what is the most probable conformation to be used as starting coordinates?’. The ^1^ *C*_4_ conformer has the large sulfate group in the less-preferred, steric clash-prone axial location, whereas the ^2^ *S*_O_ skew-boat conformer shows clear angle strain; the computed ^4^ *C*_1_ chair conformer shows little strain and has most substituents, including the sulfate, in the preferred equatorial location. However, we know that the cyclization reaction locks l-sugars, at least initially, in a ^1^ *C*_4_ conformation (Fig. 3 ▸, southern hemisphere in the Cremer–Pople diagram), and the sugar is not going to traverse any south-to-north conformational itinerary without enzymatic intervention, as the energetic penalty would exceed the final benefit, which would be in the region of 2 kcal mol^−1^ as estimated by *RDKit*, by an order of magnitude (Davies *et al.*, 2012 ▸). Thus, sampling conformations in torsional space can help to find a global energy minimum, but one that might not be attainable in nature. Similarly, using a solution NMR structure as a model might prove an even worse choice, as this technique is able to capture snapshots of dynamic transitions and these are unlikely to be representative of crystalline molecule populations. To date all occurrences of IDS within PDB entries solved crystallographically at atomic resolution (better than 1.5 Å) have the ring in the ^1^ *C*_4_ chair conformation. May this cautionary tale serve to highlight why including experimentally determined and manually curated small-molecule structures in monomer dictionaries (as the PDB is currently doing in collaboration with the Cambridge Crystallographic Data Centre; CCDC) is essential in many of the most debatable cases.

Although monomer libraries are useful for quickly accessing restraints for the most common monomers [sulfate, various metals, glycerol, *N*-acetyl β-d-glucosamine (a sugar) and the haem cofactor cover the top ten], they cannot contain information for every compound. Working with a new monomer involves generating a dictionary from its chemical definition. If a SMILES string is not available [for example C(C(CO)O)O for glycerol, GOL in the PDB_CCD_], a sketch of the molecule can be created with a number of programs: *JLigand* (Lebedev *et al.*, 2012 ▸), an improved successor to the ageing *SKETCHER* (Vagin *et al.*, 2004 ▸), is a *CCP*4 (Winn *et al.*, 2011 ▸) program written in Java which allows sketching as well as the generation of covalent bonds, for example the N-glycosidic bond in protein N-glycosylation, between a newly created or imported monomer and the protein; the *Ligand Builder* tool within *Coot* (Emsley *et al.*, 2010 ▸) is a free, universally accessible program that combines a familiar interface with powerful functionality and has been integrated as the default sketching tool in the *CCP*4*i*2 (the new *CCP*4 graphical interface) ligand-creation task; the *PRODRG* web server (Schüttelkopf & van Aalten, 2004 ▸) offers a Java applet for creating chemical diagrams and is directly integrated with the restraint-generation backend; although they are general-purpose chemical sketching tools, *ChemDraw* (Evans, 2014 ▸) and *Marvin* (ChemAxon) offer the possibility of creating a .mol file which can be read by most dictionary generators.

Finally, restraints and starting coordinates must be produced from the molecular definition before the model can be built and refined in an interactive graphics program such as *Coot* (Emsley *et al.*, 2010 ▸). This process has been reviewed in depth by Steiner & Tucker (2017 ▸), but for the sake of completeness its application to the creation of a dictionary for a cyclic monosaccharide will be demonstrated and discussed here. Some modern dictionary-generation software is capable of generating energy-minimized conformers, and their functionalities can be summarized as follows. *ACEDRG *(Long *et al.*, 2017 ▸) is a new *CCP*4 tool that has been designed to fulfil a twofold purpose: mining structural resources such as, but not restricted to, the Crystallography Open Database (COD; Gražulis *et al.*, 2009 ▸, 2012 ▸), and creating dictionaries using knowledge derived from these resources. *ACEDRG* uses *RDKit* for generating conformers *via* torsional exploration and minimization but, as pointed out above, might produce unexpected results. A somewhat older and thus further developed program, *eLBOW* (Moriarty *et al.*, 2009 ▸) from the *PHENIX* suite (Adams *et al.*, 2010 ▸, 2011 ▸) can use a number of methods for deriving the restraints and creating and optimizing the conformer: applying a simple force field, using semi-empirical methods such as AM1 (Dewar *et al.*, 1985 ▸) or running full quantum-chemical calculations with *GAMESS* (Schmidt *et al.*, 1993 ▸), although this requires securing a separate, cost-free licence. As the next program down the list, it offers the possibility of obtaining restraint information from CCDC *Mogul* (Bruno *et al.*, 2004 ▸). *Grade* (Smart *et al.*, 2014 ▸), available as a standalone program and as a freely accessible web server, is a companion tool to the refinement software *BUSTER*/*TNT* (Blanc *et al.*, 2004 ▸) that also uses CCDC *Mogul* for deriving restraints and, as does *eLBOW*, can rely on semi-empirical methods for those cases for which *Mogul* does not offer any data. Finally, the *PRODRG* server (Schüttelkopf & van Aalten, 2004 ▸) relies on the *GROMACS* package (Van Der Spoel *et al.*, 2005 ▸) for both energy minimization and restraint generation.

**Figure 8:**
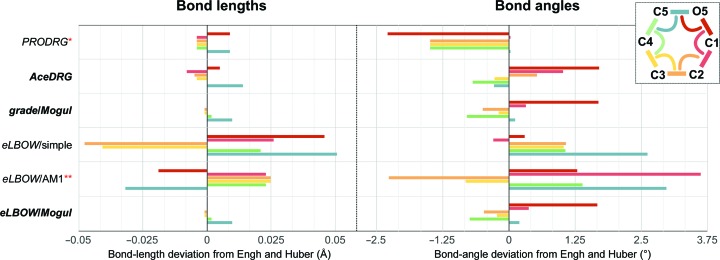
Generating a dictionary for α-d-glucopyranose from a SMILES string. Bond and angle geometries have been colour-coded according to the top-right inset panel. Horizontal lines represent deviations from Engh & Huber (1991 ▸). The three methods showing the closest agreement are shown in bold: *ACEDRG*, *grade* using *Mogul*, and *eLBOW* using *Mogul*. Red asterisks: *PRODRG* and *eLBOW* using the AM1 method did not obtain the lowest-energy conformation (^4^ *C*_1_ for d-glucose) as starting coordinates, and *PRODRG* produced the incorrect absolute configuration, turning d-glucose into its C5-epimer l-idose. *Mogul* (Bruno *et al.*, 2004 ▸) is the current geometric target that the PDB are using as validation for hetero compounds.

The results obtained by using these programs on the O[C@@H]1[C@H](O)[C@@H](O)[C@H](O)[C@]([H])(O1)(CO) SMILES string (α-d-glucopyranose; GLC in the PDB_CCD_) with default parameters are compared with Engh and Huber geometry (Engh & Huber, 1991 ▸) in Fig. 8 ▸. This SMILES string, which is different from that in the PDB_CCD_, can produce standard atom names (C1/O1…C6/O6) if processed in the expected left-to-right order. To keep it simple yet informative, only endocyclic bonds are shown, as these are expected to differ most. It becomes evident from the comparison that whereas Engh and Huber assigned fixed values to bonds and angles, other, newer methods expect these to be affected by the context (see the uniform result for *PRODRG*’s angular deviations from Engh and Huber). Also, those methods based on context-sensitive mining of small-molecule databases (*ACEDRG*, *grade* and *PHENIX eLBOW* using *Mogul*) are those that show the closest agreement, as judged by their similar deviation profiles. Finally, *PRODRG*, and *PHENIX eLBOW* using the AM1 method, did not arrive at the expected ^4^ *C*_1_ conformation, with the former getting the wrong absolute configuration, thus turning d-glucose into its C5-epimer l-idose. In order to rule out any problems with the processing of the SMILES string, the code from the PDB_CCD_ GLC entry was also tried, arriving at an identical result.

The starting coordinates in a user-generated dictionary, very much like those in the existing PDB_CCD_ entries, should always reflect the most probable, minimal energy conformation. For pyranosides, this is essentially a known parameter: rigid chair conformations in most structures unless there are any *sp* ^2^-hybridized C atoms forming endocyclic (see 4AM in the PDB_CCD_) or exocyclic (see 149 in the PDB_CCD_) double bonds, thus unsaturating the ring. Bulky or electron-dense substituents are known to have an effect on ring puckering (how much atoms move away from the mean ring plane) owing to steric effects, but these are unlikely to force a different conformation, as discussed above. At higher resolution (better than 1.5 Å), where geometric restraints are downplayed in favour of experimental terms (Steiner & Tucker, 2017 ▸), the electron density should always narrow the number of possible conformations of a ligand down to a couple at most. However, at the other end of the spectrum, lower resolution diffraction data sets, which are more affected by solvent contribution and cross-crystal movements and defects, often lead to the synthesis of less featureful or incomplete maps for the sugars. Such maps are typically uninformative for ascertaining the conformation of a monosaccharide, and the comprehensive set of restraints that dictionaries contain may not suffice to force an initial distorted model into the most probable conformation.

### 4.1. In practical terms   

Monomer libraries should typically contain the minimal energy conformation for the starting coordinates, which for sugars will be one of the two possible chairs. This must also be ensured when generating dictionaries for new sugars. There are various methods for calculating such minimal energy conformations, and most restraint-generation programs provide one or a few methods. Those programs based on context-sensitive mining of small-molecule databases show the closest agreement. As the ligand-validation task force have recently agreed (Adams *et al.*, 2016 ▸), such databases provide the best available predictive power and are particularly well suited for the validation of molecular geometries. Consequently, any restraint target that wildly differs from what the programs recommended above produce will, most critically when refining against low-resolution data, systematically generate geometric outliers upon validation and deposition. This final point will be reiterated in the last section.

## 5. Modelling and refinement   

Initially, the PDB encoded both anomeric configurations into a single three-letter code. Consequently, refinement programs had to rely on MODRES records to rename each residue and point to the correct set of restraints. The PDB archive was then remediated (Henrick *et al.*, 2008 ▸) and the PDB_CCD_ now holds independent three-letter codes for each anomer (see Table 2 ▸ for the correspondence between IUPAC long and short names and the PDB_CCD_ notation), making the renaming process unnecessary. While most of the sugars appear to be fine, β-d-xylose (XYP), a sugar that is central to plant biology, still does not follow the same standard atom-naming convention. This issue has caused problems downstream, as programs operating on the PDB_CCD_ definition may not recognize this entry as a sugar. Such is the case with *LIBCHECK* (Vagin *et al.*, 2004 ▸), which was used to generate the CCP4 monomer library: indeed, this entry is classified as a ‘non-polymer’ instead of ‘pyranose’, and therefore *REFMAC*5 (Murshudov *et al.*, 2011 ▸) is unable to detect glycosidic type links between XYP and any other sugar, including XYP. Other, potentially unrelated issues that *LIBCHECK* has with sugars include the generation of one 0° endocyclic torsion which keeps four ring atoms coplanar and therefore imposes the wrong envelope or half-chair conformations. *Privateer* (Agirre, Iglesias-Fernández *et al.*, 2015 ▸) will report any incorrect torsions found in the library if run from the command line. The problem is known to affect at least 60 sugar entries in the *CCP*4 monomer library, including NAG, BGC and BMA. These problems, along with the fact that the geometry target that *LIBCHECK* produces is consistent with Engh & Huber (1991 ▸), which is now inconsistent with the new context-dependent geometries, highlight the need for a regeneration of the whole library using a modern tool such as *ACEDRG* (Long *et al.*, 2017 ▸).

### 5.1. Building a model   

The very first step after obtaining a sugar from the monomer library is fitting it to electron density, and this should only be attempted when the rest of the structure is well refined (refer to the next section for more details). At higher resolution, the electron density for O atoms often becomes clearly higher than that for C atoms and therefore hints at where the endocyclic O atom should be. Sugar residues can be manually rotated and translated in *Coot* (Emsley *et al.*, 2010 ▸) until the location and orientation is roughly correct, and then automatically fitted to the map on an individual basis using the real-space refinement tool of the program. Alternatively, and also within *Coot*, the ‘jiggle fit’ function may be able to determine the best orientation of a sugar model automatically, although this might require several trials. Once all sugars in an oligosaccharide have been fitted to density, the leaving groups, which should now superpose on the hydroxyl group of the following sugar, must be deleted in order to subsequently form the links. Sugar monomers should then be renumbered and moved to the same chain and model. Those sugars numbered sequentially will be treated as linked in *Coot* and thus their linkages can be refined with the ‘sphere refinement’ function of the program (accessible from the toolbar). However, torsion restraints may need to be manually activated when working with low-resolution data or incomplete maps. Lowering the weight for the experimental terms may also help in retaining the lowest-energy conformations of the sugars. These can be enforced by using aperiodic torsion restraints (previously referred to in the literature as monoperiodic), which can be produced with *Privateer* (Agirre, Iglesias-Fernández *et al.*, 2015 ▸) upon analysing a structure with sugars in higher-energy conformations. An aperiodic torsion restraint enforces a single torsional value, and by applying a set of torsion values to the ring bonds, it is possible to enforce one particular conformation. This has been shown to work well in a number of examples (Agirre *et al.*, 2016 ▸; Gudmundsson *et al.*, 2016 ▸; Agirre, Iglesias-Fernández *et al.*, 2015 ▸).

**Figure 9:**
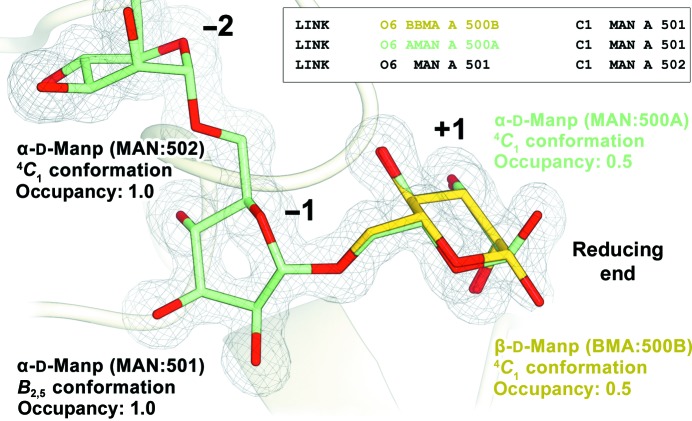
Glycosidic bonds, distortion in the −1 subsite and mutarotation at the reducing end. The figure shows the active site of an α-mannanase enzyme reported by Thompson *et al.* (2015 ▸, 2016 ▸), which was crystallized in complex with α1–6-mannopentaose. Sugars have been numbered according to standard practice, from 500A (and its alternate configuration, 500B) at the reducing end to 504 (not shown) at the nonreducing end. LINK records can be defined as shown in the inset (only the part relevant to residue identification is shown; see the PDB format specification for the full syntax) and have to be replicated to link both configurations of residue 500, which in turn have their respective occupancies reduced to 0.5. The sugar in the −1 subsite (nomenclature defined in Davies *et al.*, 1997 ▸) is distorted by the catalytic residues (not shown) to a *B*_2,5_ conformation, which is well supported by clear electron density and described by QM/MM metadynamics simulations as part of the catalytic itinerary (Thompson *et al.*, 2015 ▸, 2016 ▸). This figure was generated with *CCP*4*mg* (McNicholas *et al.*, 2011 ▸).

LINK records can be created with *Coot* by choosing the ‘Make link…’ action from the ‘Modelling’ submenu within the ‘Extensions’ menu. These are required to explicitly link those sugars which are not consecutive, or for creating protein–sugar linkages such as the NAG_C1_–ASN_ND2_ bond in N-glycosylation or the A2G_C1_–SER_OG_ or A2G_C1_–THR_OG1_ bonds in O-glycosylation. Correct bond distances should be observed: for instance, experimental evidence suggests that the expected distance for the ASN_ND2_–NAG_C1_ bond lies in the 1.43–1.45 Å range (Mølgaard & Larsen, 2002 ▸). Covalent bonds between newly defined sugars and other entities should be defined and restrained using *JLigand* (Lebedev *et al.*, 2012 ▸). For standard bonds, which includes all glycosylations and most polysaccharides, it is possible to derive a set of standard covalent-bond definitions using reciprocal-space refinement software. The *phenix.refine* (Afonine *et al.*, 2012 ▸) graphical interface has an ‘Automatic linking options’ button under ‘Refinement settings’, which can be used to control linkage creation, although sugar–protein and sugar–sugar contacts are included by default. Proper care must be taken at this stage not to include spurious linkages caused by unexpected contacts; *REFMAC*5 (Murshudov *et al.*, 2011 ▸) can either translate existing LINK records (Fig. 9 ▸) or create linkage information based on distance on its own, richer definition of bonds, the LINKR record. These are identified by a name (*e.g.* NAG-ASN, ALPHA1-4 or BETA1-6) that references the library, which defines their chirality and geometry restraints. These will cause *REFMAC*5 to stop and write a new library to disk containing any new linkages, whether expected or not; inspecting this file is highly recommended, as it can lead to the identification of problems. Once any bad contacts have been eliminated, *REFMAC*5 should be run again on the new model. If the electron density for the sugar in the reducing end has an ambiguous shape for the C1 hydroxyl, it may be affected by mutarotation. Such a scenario can be modelled and refined using the alternate location characters of the PDB, reducing the occupancy of each instance to 0.5 and creating the LINK records between the next sugar and both anomeric forms of the mutarotated sugar (Fig. 9 ▸).

It is standard practice to number sugars from the reducing end, *i.e.* the one that has a free anomeric C atom, not linked to any other sugar. Ligand sugars may be placed in a different chain, *e.g.* ‘S’ as opposed to ‘A’, whereas glycosylation sugars have to be part of the same chain to which they are covalently linked.

Keeping minimal energy conformations during low-resolution refinement poses a challenge (Agirre, Davies *et al.*, 2015 ▸). While it is possible to reduce the weight for the experimental terms in *Coot *and perform a series of localized, highly restrained real-space refinements, this cannot be easily accomplished in reciprocal-space refinement, where there is a single weighting term operating on the whole geometric specification of the model. Particular changes tend to be of general scope, *i.e.* tightening the geometry for GLC acts on all occurrences of GLC no matter how complete or defined their electron density is. Similarly, activating torsion restraints, which to the best of the author’s knowledge are not activated by default on ligands in any refinement tool, does so on a residue-type basis. One possibility to be investigated in the future, at least for *REFMAC*5, could be the generation of localized restraints using the external restraint interface of the program, as these can act on precisely defined regions of the model. For now, the aperiodic restraints that *Privateer* generates upon detecting sugars in higher-energy conformations can be used to maintain or even fix wrong conformations (Agirre, Iglesias-Fernández *et al.*, 2015 ▸). This program generates a library in CIF format, a keyword file for activating the relevant torsions in *REFMAC*5 and scripts in Python and Scheme for loading files, activating torsion restraints and a list of outliers in *Coot*. As *CCP*4*i*2, the new graphical interface for the *CCP*4 suite, offers a follow-on job mechanism, most of these operations are performed automatically for the user. As an alternative to using torsion restraints, the *PHENIX* suite has recently included *AMBER* (Case *et al.*, 2005 ▸), which is expected to provide more realistic estimations of torsion potentials and should have a positive impact on maintaining the correct sugar conformation.

Advances are being made towards an automated interpretation of electron density in terms of sugar models: *Coot* now offers a semi-automated module for dealing with most common cases of N-linked glycosylation. This is available in the ‘Modules’ submenu under the ‘Extensions’ menu. Although not yet released publicly in binary form, the *Sails* program (Agirre & Cowtan, 2016 ▸) will offer a fingerprint-based automated detection of ligand or covalently linked sugars, with integrated validation provided by *Privateer*’s libraries. This detection technology has already been implemented successfully in other programs, such as *Nautilus* (Cowtan, 2014 ▸) and *Buccaneer* (Cowtan, 2006 ▸).

### 5.2. Modelling sugars in the active site of an enzyme   

As mentioned previously, conformational distortion in pyranosides is a rare event that, when captured in a crystal structure, usually provides a revealing snapshot of an ongoing chemical reaction. Unlikely events require conclusive experimental evidence rising above interpretational subjectivity which, even at high resolution, might play a strong role. Such is typically the case when modelling a sugar in the −1 subsite of a glycoside hydrolase (Fig. 9 ▸; for a description of the nomenclature used to identify enzyme subsites, see Davies *et al.*, 1997 ▸), where a water molecule is expected to play a role in the hydrolysis. Water molecules should always be added in the final stages of structure refinement, when phases can be expected to be most accurate, but care must be taken for them not to populate the density for the ligand. This can be accomplished by modelling the ligand pre-emptively, setting its occupancy to an arbitrarily low value, for example 0.01, so that the impact of the ligand model on phase calculation is minimized yet it does not allow waters to be fitted inside its density, and adding waters after refinement. Ligand fitting can then proceed after removal of the pre-emptively fitted fragment. The interpretation of the −1 site scenario should always be attempted first by assuming the most probable outcome: that all sugars are in the minimal energy conformation. Placing a water molecule in a density blob where the C1 hydroxyl of the sugar should be will invariably cause any refinement software to distort the conformation of the sugar in order to avoid steric clashes with the water molecule.

Negative (model) difference density might appear around the atoms of ligand sugar atoms after refinement. In such a case, the occupancies of the atoms may have to be reduced, and the ‘residue info’ option within the ‘measures’ menu in *Coot* may be used for this purpose. The real occupancy value can be approximated manually for each residue by iteratively decreasing the initial value (1.0 by default) in small amounts (*e.g.* 0.1) between refinement rounds until the *B* factors of the atoms of the sugar roughly match those of any neighbouring protein atoms establishing hydrogen bonds with it, as they can be expected to be very similar. However, this procedure is performed automatically within certain refinement programs, such as *phenix.refine* (Afonine *et al.*, 2012 ▸).

## 6. Validating, depositing and reporting a new structure   

There are three pillars in carbohydrate model validation: nomenclature, structure and conformation. Any mistakes affecting nomenclature, structure or both can lead to a distorted ring, incorrect bond conformations or both. Higher-energy ring or bond conformations do not necessarily spawn from previous mistakes introduced during model building, but can result from refining a model against low-resolution data with fewer restraints than required. Such problems, which span across all refinement programs, were highlighted recently using N-glycan-forming d-pyranosides as a subject study (Agirre, Davies *et al.*, 2015 ▸).

### 6.1. Nomenclature   

Stereochemistry, including anomeric and absolute configurations, is embedded in the three-letter codes from the PDB_CCD_. Chirality restraints, which will be used for validation upon deposition, are tied to these codes, so proper care must be ensured when choosing the relevant code (Table 2 ▸). Bonds between sugars must be produced according to IUPAC guidelines, erasing the leaving groups and generating a LINK record between the anomeric C atom (C1 for most aldoses, C2 for most ketoses) and the linked substituent (*e.g.* O4 in Fig. 4 ▸). Bond and residue nomenclature can be validated with the *PDB-CARE* server (Lütteke & von der Lieth, 2004 ▸) and as part of the structure-solution process with *Privateer* (Agirre, Iglesias-Fernández *et al.*, 2015 ▸). When depositing a crystal structure containing a mixture of anomers owing to muta­rotation at the reducing end of the polysaccharide chain (Fig. 9 ▸), both alternate configurations, which the PDB will refer to as conformations, even though they represent a configurational change, must have the appropriate three-letter code (*e.g.* BMA and MAN in Fig. 9 ▸). These special cases can be validated with *Privateer* (Agirre, Iglesias-Fernández *et al.*, 2015 ▸).

It is important that any sugar–sugar and sugar–protein bonds are explicitly declared upon deposition using the PDB LINK records, and that these show the expected distance. The PDB will otherwise perform chemistry perception on an incomplete model (*e.g.* lacking H atoms), and may conclude that sugars are deoxy, *i.e.* showing two H atoms instead of one linked to the (endocyclic) C1. An example of this potential substitution is having NAG (*N*-acetyl β-d-glucosamine) renamed 5AX (deoxy derivative). Another potential substitution may occur when depositing N-glycosylation showing an incorrect α ASN–NAG bond; the PDB will detect this, offer a substitution for NDG (the less likely, α-anomeric configuration of NAG) and add a caveat to the entry, as N-glycosylation is always β and thus it is very likely the depositors have modelled it incorrectly. Depositors should hold off deposition until such conflicts are resolved, which will involve fixing the atomic model and resubmitting the coordinates.

Regardless of the internal conceptual reduction that the PDB may perform on oligosaccharide entities, for example turning disaccharides such as cellobiose into β-d-gluco­pyranosyl-(1–4)-β-d-glucopyranose, *i.e.* two BGC entities, they will retain whatever chemical entity was reported by the depositor. However, as computational mining efforts most typically operate on monosaccharide entities, there is a case for defining oligosaccharide structures using individual monosaccharide three-letter codes.

**Figure 10:**
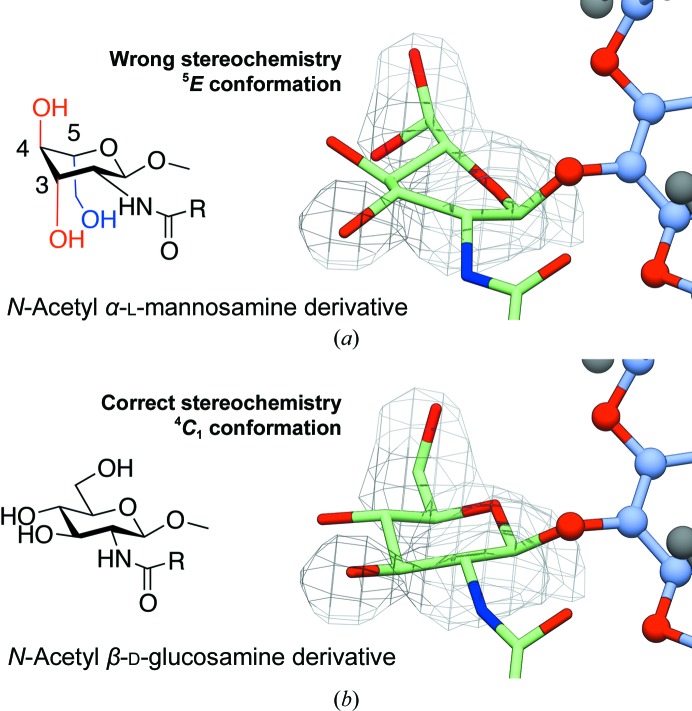
Conformational validation. (*a*) Chemical errors in the key TM9 sugar, deposited as an *N*-acetyl α-l-mannosamine derivative (left, PDB entry 4k3t, now superseded by PDB entry 5awv), and their impact on the published structure (right). (*b*) Correct stereochemistry (left) and re-refined structure after correcting the errors (right). Re-refining the structure with the correct stereochemistry (*N*-acetyl β-d-glucosamine derivative) causes the sugars to end up in the minimal energy chair conformation. For the stereochemically correct ligand, OMIT density maps (*mF*_o_ − *DF*_c_ coefficients, contoured at 2σ) show plausible density for the putative diol intermediate at least in chains *M* and *N*. While the maps selected by the original authors may not be too different from those obtained through refinement of the correct chemical species at the C6 diol, publishing a distorted sugar with the wrong stereochemistry at almost every position casts legitimate doubt on their glyco-chemical conclusions. This figure was generated with *CCP*4*mg* (McNicholas *et al.*, 2011 ▸).

Depositing a new carbohydrate definition in the PDB_CCD_ only requires that the PDB understands the chemistry of the ligand and is able to rationalize it using available software. No cross-checks are performed between the reported and the deposited chemistries. In a recent example, the TM9 entry of the PDB_CCD_ was reported to be an *N*-acetyl β-d-glucosamine derivative showing a diol intermediate in an addendum (Liu *et al.*, 2015 ▸) to the original publication (Liu *et al.*, 2011 ▸), which apparently had caused controversy. Despite being welcomed in the accompanying editorial as the outcome of a constructive community self-scrutiny, the deposited ligand structure (PDB entry 4k3t, now superseded by PDB entry 5awv) showed three incorrect chiralities, including the absolute configuration of the sugar (Fig. 10 ▸). As a result of this considerable mismatch between the modelled ligand and the electron density, which indeed hinted at glucose stereochemistry, the model ended in higher-energy conformations across the whole crystal structure in all four chains in the asymmetric unit. These errors are easily identified using *Privateer* (Agirre, Iglesias-Fernández *et al.*, 2015 ▸). After a hiatus, the authors corrected this mistake and deposited an amended structure with the affected sugars, but not the rest, showing the expected ^4^ *C*_1_ conformation (PDB entry 5awv). This is an example of the vicious circle that incorrectly defined new sugars, and ligands in general, can cause: idealized (but wholly incorrect) coordinates for the TM9 entry calculated from the flawed chemical description of the depositors are available from the PDB_CCD_. Before PDB entry 4k3t was retracted, the description of the compound was hyperlinked to a structure, which in turn pointed to the original publication, where it was presented as something totally different. Anyone, whether in the antimicrobial field or beyond, using the idealized, but misleading, coordinates from the PDB_CCD_ (based upon the deposition) will simply propagate the errors.

### 6.2. Structure   

At high resolution, most problems related to the structure of a glycan/oligosaccharide after refinement will result in conformational problems that can be detected and tackled at the monosaccharide level with *Privateer* (Agirre, Iglesias-Fernández *et al.*, 2015 ▸). At lower resolution, it is of the utmost importance that structures either conform to prior glyco-chemical knowledge or have accompanying experimental evidence, for example mass spectrometry, thin-layer chromatography or high-pressure chromatography using fluorescently labelled sugars, that supports any unusual stereochemistry or linkage. A recent example of such a situation is the structure of a sialylated IgG Fc fragment reported by Crispin *et al.* (2013 ▸), with PDB accession code 4byh. The sialic acid at the end of the 6-arm was reported to have a high average *B* factor (131.5 Å^2^) with respect to the rest of the glycan (87.1 Å^2^, at a reported resolution of 2.3 Å) and was modelled in scarce density. However, a routine experimental technique such as HPLC can be combined with fluorescent labelling of a target monosaccharide (Neville *et al.*, 2004 ▸), providing sufficient evidence for the presence of otherwise elusive terminal sugars, as the work by Crispin and coworkers testifies. Indeed, Crispin *et al.* (2007 ▸) had previously advocated that the criterion described in this paragraph should become standard practice whenever electron density offers a far from conclusive answer.

While *Privateer* (Agirre, Iglesias-Fernández *et al.*, 2015 ▸) and *PDB-CARE* (Lütteke & von der Lieth, 2004 ▸) will cross-check input structures against the common characteristics of glycans based on the expression system used, it is worth using a database such as UniCarbKB (Campbell *et al.*, 2014 ▸) or glycosciences.de (Loss & Lütteke, 2015 ▸; Lütteke *et al.*, 2006 ▸) through *GlycanBuilder* (Damerell *et al.*, 2012 ▸, 2015 ▸) to obtain experimental confirmation for longer or more complex glycans. A recent summary of the available tools has been published by Emsley *et al.* (2015 ▸).

When working with a low-resolution structure, it might be necessary to tighten the geometry of pyranose residues in order to prevent any conformational deviations. Matching a geometry target very closely can result in hundreds of bond-length and bond-angle outliers upon deposition if the target used does not agree with what the PDB are using for validation, which at the time of this review is *Mogul* (Bruno *et al.*, 2004 ▸). In such cases, using Fig. 8 ▸ as guidance for choosing a dictionary generator is advised.

### 6.3. Conformation   

Pyranoside high-energy conformations are so rare that their occurrence should be reported as an exceptional event, much in the way that torsional (Ramachandran) outliers are listed in the data-statistics table of a crystallographic experiment. This was originally suggested by Stewart-Jones *et al.* (2016 ▸), later proposed by Agirre *et al.* (2016 ▸) and recently adopted by Gudmundsson *et al.* (2016 ▸).

**Figure 11:**
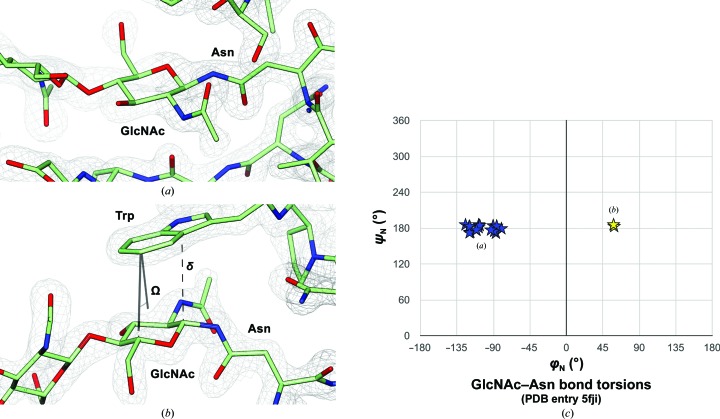
Glycosidic bond torsions can be affected by stacking interactions. (*a*) The most frequent conformation of the GlcNAc–Asn bond as found by Imberty & Perez (1995 ▸) and Lütteke *et al.* (2005 ▸), plotted as blue stars in (*c*) for PDB entry 5fji. (*b*) This flipped conformation of GlcNAc lies in a secondary torsional energy minimum that was originally described by Imberty & Perez (1995 ▸), and is stabilized by a stacking interaction with a neighbouring tryptophan, the character of which is conserved across homologues in order to maintain the conformation of this bond (Agirre *et al.*, 2016 ▸). Stacking interactions can be computed with *Privateer* (Agirre, Iglesias-Fernández *et al.*, 2015 ▸), using the definition proposed by Hudson *et al.* (2015 ▸), which states that δ must be shorter than 4.0 Å and the Ω angle must be smaller than 30°. (*c*) Ramachandran-like plot calculated with *Privateer* using the convention from Lütteke (2009 ▸), also depicted here in Fig. 5 ▸. This figure was generated with *CCP*4*mg* (McNicholas *et al.*, 2011 ▸).

Bond torsions can be analysed with *Privateer* (Agirre, Iglesias-Fernández *et al.*, 2015 ▸) and also compared with existing structures using the *CARP* server (Lütteke *et al.*, 2005 ▸). Certain bond conformations can be favoured by the presence of neighbouring aromatic residues (Fig. 11 ▸), as reported by Agirre *et al.* (2016 ▸). Although existing structures provide valuable information in terms of preferred bond conformations, the underlying structural data should be curated by minimal energy ring conformation in the future in order to eliminate misleading data points, as distorted ring conformations have a clear impact on how and where the substituents are placed, thus strongly affecting the reported linkage conformation.

## 7. Concluding remarks   

The computational side of structural glycobiology is slowly catching up with the rest of the field. For validation methods to succeed in preventing many of the mistakes mentioned above, they have to be integrated much more closely into the structure-determination process. Web services, while being generally easy to use and requiring a setup as simple as a browser, represent an unsurmountable barrier for confidential projects, and even in nonconfidential ones they tend to occupy a residual, often overlooked, step at the end of such process.

Currently, the accuracy with which protein models are determined from low-resolution data sets is, thanks to a new generation of context-dependent restraints (Moriarty *et al.*, 2014 ▸, 2016 ▸; Tronrud & Karplus, 2011 ▸; Tronrud *et al.*, 2010 ▸), much higher than that of carbohydrates (Agirre, Davies *et al.*, 2015 ▸). With cryo-EM now routinely venturing into the 2.0–4.0 Å resolution range, it is becoming increasingly clear that sugar chemistry will need to find its way into the current refinement methods: new dictionaries will have to be produced with accurate torsion restraints, force fields may have to be introduced in order to keep conformations and contacts within chemical expectations, and new combined validation approaches will be needed to assess and support distortion in active sites.

## Figures and Tables

**Table 1:** Excerpt from a crystallographic table reporting the structure of a glycoprotein Proposal for the presentation of pyranose conformational data. These results were computed using *Privateer* (Agirre, Iglesias-Fernández *et al.*, 2015 ▸) on PDB entry 4iih, a heavily glycosylated fungal glycosyl hydrolase structure reported by Suzuki *et al.* (2013 ▸). Taking into account the resolution that this structure was determined at (2.0 Å), all pyranose sugars should have been restrained to show a ^4^ *C*_1_ conformation, as the experimental data do not offer sufficient evidence to support higher-energy conformations, just as Ramachandran outliers have been kept to a minimum (only one in 1657 residues analysed, as shown in the PDB validation report).

Pyranose conformations† (total/percentage)	
Lowest energy conformations	80/90.91
Higher energy conformations	8/9.09

† Calculated using *Privateer* (Agirre, Iglesias-Fernández *et al.*, 2015 ▸) from the *CCP*4 suite (Winn *et al.*, 2011 ▸), and presented as introduced by Agirre *et al.* (2016 ▸).

**Table 2:** Correspondence between IUPAC nomenclature and PDB_CCD_ notation for the most frequently deposited sugars Pyranose forms are assumed unless an explicit indication is given (*e.g.* α-L-arabinofuranose).

Short name	Complete name	PDB_CCD_ notation
Glc	α-D-Glucose	GLC
	β-D-Glucose	BGC
Gal	α-D-Galactose	GLA
	β-D-Galactose	GAL
Man	α-D-Mannose	MAN
	β-D-Mannose	BMA
Fuc	α-L-Fucose	FUC
	β-L-Fucose	FUL
Xyl	α-D-Xylose	XYS
	β-D-Xylose	XYP
Ara	α-L-Arabinopyranose	ARA
	α-L-Arabinofuranose	AHR
Fru	β-D-Fructofuranose	FRU
Rib	α-D-Ribofuranose	RIB
	β-D-Ribofuranose	BDR
GlcN	α-D-Glucosamine	PA1
	β-D-Glucosamine	GCS
GlcA	α-D-Glucuronic acid	GCU
	β-D-Glucuronic acid	BDP
GalA	α-D-Galacturonic acid	GTR
	β-D-Galacturonic acid	ADA
ManA	α-D-Mannuronic acid	MAV
	β-D-Mannuronic acid	BEM
IdoA	α-L-Iduronic acid	IDR
GlcNAc	*N*-Acetyl α-D-glucosamine	NDG
	*N*-Acetyl β-D-glucosamine	NAG
GalNAc	*N*-Acetyl α-D-galactosamine	A2G
	*N*-Acetyl β-D-galactosamine	NGA
ManNAc	*N*-Acetyl α-D-mannosamine	BM3
Neu5Ac	5-*N*-Acetyl α-D-neuraminic acid	SIA
	5-*N*-Acetyl β-D-neuraminic acid	SLB
Kdn	Keto-deoxy α-D-nonulonic acid	KDM
	Keto-deoxy β-D-nonulonic acid	KDN
